# Serum Metabolites Differentiate Amnestic Mild Cognitive Impairment From Healthy Controls and Predict Early Alzheimer's Disease via Untargeted Lipidomics Analysis

**DOI:** 10.3389/fneur.2021.704582

**Published:** 2021-08-02

**Authors:** Lumi Zhang, Lingxiao Li, Fanxia Meng, Jie Yu, Fangping He, Yajie Lin, Yujie Su, Mengjie Hu, Xiaoyan Liu, Yang Liu, Benyan Luo, Guoping Peng

**Affiliations:** ^1^Department of Neurology, First Affiliated Hospital, Zhejiang University School of Medicine, Hangzhou, China; ^2^Department of Neurology, Saarland University, Homburg, Germany

**Keywords:** Alzheimer's disease, amnestic mild cognitive impairment, untargeted lipidomics, serum, cholesteryl ester, ganglioside GM3, neuromedin B

## Abstract

**Background and Aim:** Alzheimer's disease (AD) is the most common type of dementia and presents with metabolic perturbations early in the disease process. In order to explore biomarkers useful in predicting early AD, we compared serum metabolites among patients suffering different stages of AD.

**Methods:** We recruited 107 participants including 23 healthy controls (HC), 21 amnestic mild cognitive impairment (aMCI), 24 non-amnestic mild cognitive impairment (naMCI) and 39 AD patients. Via liquid chromatography-mass spectrometry based serum untargeted lipidomics analysis, we compared differences in serum lipid metabolites among these patient groups and further elucidated biomarkers that differentiate aMCI from HC.

**Results:** There were significant differences of serum lipid metabolites among the groups, and 20 metabolites were obtained under negative ion mode from HC and aMCI comparison. Notably, 16:3 cholesteryl ester, ganglioside GM3 (d18:1/9z-18:1) and neuromedin B were associated with cognition and increased the predictive effect of aMCI to 0.98 as revealed by random forest classifier. The prediction model composed of MoCA score, 16:3 cholesteryl ester and ganglioside GM3 (d18:1/9z-18:1) had good predictive performance for aMCI. Glycerophospholipid metabolism was a pathway common among HC/aMCI and aMCI/AD groups.

**Conclusion:** This study provides preliminary evidence highlighting that 16:3 cholesteryl ester were useful for AD disease monitoring while ganglioside GM3 (d18:1/9z-18:1) and neuromedin B discriminated aMCI from HC, which can probably be applied in clinic for early predicting of AD.

## Introduction

Alzheimer's disease (AD) is the commonest type of dementia and presents with a wide range of metabolic perturbations early in the disease process (1). However, there is currently no effective treatment for AD, although diagnosis and treatment should commence as early as possible in the disease course. It is therefore particularly important to identify AD in its preclinical stage.

*In vivo* detection of senile plaque positron emission tomography and cerebrospinal fluid (CSF) Aβ42/tau level testing are not universally feasible for AD detection due to high cost and procedure invasiveness. The accessibility and cost-effectiveness of blood-based biomarkers, however, make them rather suitable for clinical use and especially so for disease surveillance (2). In recent years, a number of metabolomics studies have attempted to obtain from serum, plasma or whole blood specimens biomarkers useful for predicting AD. Biomarkers currently most recognized include the Aβ42:Aβ40 plasma ratio as well as blood neurofilament light chain, plasma phosphorylated tau 181 and phospho-tau 217 levels (3–7). Barupal et al., reported that monounsaturated lipid metabolism plays a role in early AD, whereas polyunsaturated lipid metabolism was more relevant to later stages of AD (8). Kim et al., suggested that triacylglycerol 50:1, diacylglycerol 18:1/18:1 and phosphatidylethanolamine 36:2–when incorporated with test scores of common measurements of cognitive impairment–improve selectivity in identifying mild cognitive impairment (MCI) (9). Most of the aforementioned researches, however, involved comparisons among AD and healthy control (HC) groups, with or without MCI groups. Where present, MCI groups were seldom subdivided further. In terms of the major areas of cognitive impairment, MCI can be subdivided into amnestic mild cognitive impairment (aMCI) and non-amnestic mild cognitive impairment (naMCI) (10). Previous studies have shown that aMCI has a higher tendency to progress to AD (11), while naMCI more easily progresses into other types of dementia, such as vascular or Lewy body dementias (12). Here, we subdivided MCI patients into aMCI and naMCI subgroups for further analysis and utilized liquid chromatography-mass spectrometry based serum untargeted lipidomics analysis to obtain raw data. This study aimed to determine whether there was any difference in serum metabolites among HC, MCI, and AD groups, and to elucidate potential biomarkers for predicting early AD.

## Methods and Materials

### Subjects

A total of 107 elderly patients were recruited from the memory clinic of the First Affiliated Hospital, Zhejiang University School of Medicine between October 2016 and June 2018. This included 23 HC, 45 MCI patients (21 aMCI and 24 naMCI patients), and 39 cases of AD. Patients included in this study were diagnosed as probable AD according to the criteria of the National Institute of Neurological and Communicative Disorders and the Stroke and Alzheimer Disease and Related Disorders Association (13). Patients suffering MCI also met the Petersen criteria (14). Diagnostic criteria for aMCI were: (1) memory loss confirmed by a relative or friend; (2) auditory verbal learning test delayed recall score <1.5 standard deviations as compared to age and education matched control subjects; (3) other cognitive functions relatively intact; mini-mental state examination (MMSE) ≥ 24 or Mattis Dementia Rating Scale ≥ 120 (in the case of junior high school and above education levels); (4) preserved ability to work and socialize and with activities of daily life either not affected or only affected by memory loss; (5) failure to meet diagnostic criteria for dementia (15). Patients without any signs of cognitive impairment as determined by cognitive assessment scales (A total of 11 scales for testing memory, attention, executive ability, visual space, etc.) or neuropathological changes noted on magnetic resonance imaging were included as HC. Written informed consent were collected from all the subjects prior to participating in the study, in accordance with protocols approved by the Ethics Committee of the First Affiliated Hospital of Zhejiang University School of Medicine (reference number: 2016-315).

### Serum Sample Collection and Processing

Patients with co-morbidities including thyroid disease, diabetes and other neuropsychiatric illnesses were excluded from this study. All selected patients were either not treated or relevant drugs were stopped 1 month prior to blood sample collection. All blood samples were collected on an empty stomach and promptly placed in 5 mL vacutainer tubes. After centrifugation, serum was separated and stored at −80°Cfor later use. Serum samples (40 μL) were subsequently added to corresponding 300 μL 96-well plate wells of centrifuge tube racks; 120 μL of isopropanol was added to the wells and the plate was shaken for 1 min, kept at room temperature for 10 min, and refrigerated at −20°C overnight. Samples were centrifuged the following day at 4,000 g at 4°C for 20 min. Next, 25 μL of supernatant and 225 μl of isopropanol:acetonitrile:water (2:1:1) solution were mixed in a new 300 μL 96-well plate for dilution. To monitor instrument analysis and test-retest reliability, quality control samples were prepared by pooling 20 μL of each sample and analyzing these together with other samples. These quality control samples were analyzed every 10 samples.

### Liquid Chromatography-Mass Spectrometry Analysis

All chromatographic separations were performed using an ultra-performance liquid chromatography system (Waters, UK). An ACQUITY ultra-performance liquid chromatography CSH C18 column (100 mm × 2.1 mm; 1.7 μm, Waters, UK) was used for separation. The column oven was maintained at 55 °C. The flow rate was 0.4 ml/min and the mobile phase consisted of solvent A [ACN:H2O (60:40), 0.1% formatae and 10 mM ammonium formate] and solvent B [IPA:ACN (90:10), 0.1% formate and 10 mM ammonium formate]. Gradient elution conditions were set as follows: 0–2 min, 40–43% phase B; 2.1–7 min, 50–54% phase B; 7.1–13 min, 70–99% phase B; 13.1–15 min, 40% phase B. Injection volume for each sample was 10 μL.

**Figure 1:**
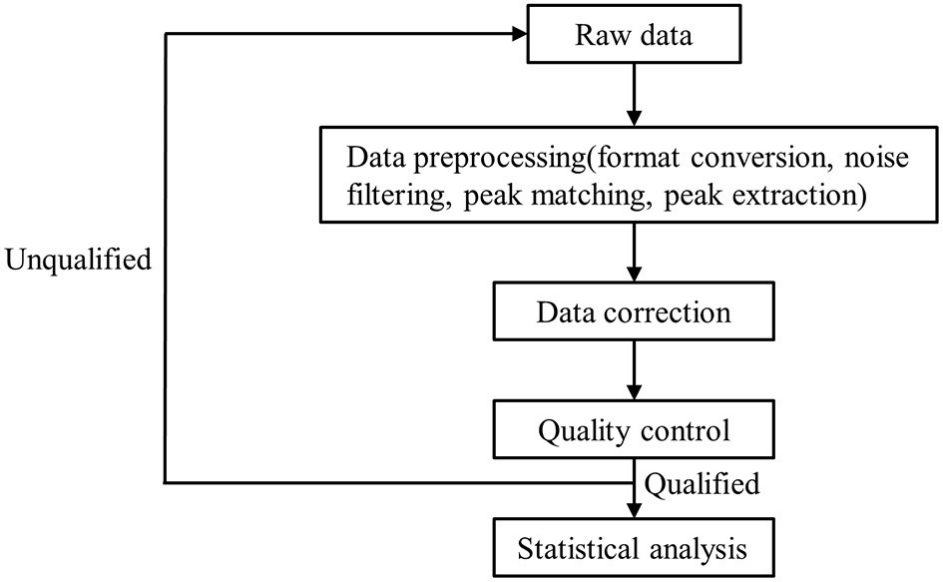
The flow chart of raw data processing.

A high-resolution tandem mass spectrometer, Xevo G2 XS QTOF (Waters, UK), was used to detect metabolites eluted from the column; Q-TOF was operated in both positive and negative ion modes. For the positive ion (POS) mode, the capillary and sampling cone voltages were set at 3.0 kV and 40.0 V, respectively. For the negative ion (NEG) mode, the capillary and sampling cone voltages were set at 2 kV and 40 V, respectively. Mass spectrometry data were acquired in centroid MSE mode. The TOF mass ranged from 100 to 2,000 Da and 50 to 2,000 Da in positive and negative modes, respectively, and the survey scan time was 0.2 s. For MS/MS analysis, all precursors were fragmented using 19–45 eV; scan time was also 0.2 s. During acquisition, the LE signal was acquired every 3 s to calibrate mass accuracy. In order to evaluate the stability of liquid chromatography-mass spectrometry throughout acquisition, a quality control sample (pool of all samples) was acquired after every 10 samples. Raw data were processed as detailed in Figure 1. The raw data from the mass spectrometer is imported into the commercial software Progenesis QI (version 2.2, hereinafter referred to as QI) for peak extraction to obtain MS1, MS2, retention time, and ion area information. The metabolite identification is based on the databases HMDB and LipidMaps (Supplementary Tables 1, 2).

### Pattern Recognition Analysis

After normalization and integration via support vector regression, processed data were uploaded into MetaboAnalyst (www.metaboanalyst.ca) for further analysis (16–18). Principal component analysis (PCA) and orthogonal partial least squares-discriminant analysis (OPLS-DA) models were established using SIMCA-P 14.1 (Umetrics, Umea, Sweden); these models were used to analyze data collected from both positive and negative models after logistic transformation and Pareto scaling. Univariate analysis included use of the Student's *t*-test and variable fold-change analysis.

### Statistical Analyses

Statistical analyses were performed using MetaboAnalyst and SPSS v26.0 (IBM, USA). One-way ANOVA and chi-squared test were used to compare differences in demographic characteristics and serum metabolites among HC, MCI, and AD groups; PCA, PLSDA, dendrogram, and heatmap analyses were performed for the obtained differential metabolites. Differential serum metabolites of aMCI and HC groups were analyzed on the MetaboAnalyst website and subjected to further analysis, such as OPLS-DA and volcano map construction. We build multiple models and screened the optimal model for prediction (see Supplementary Table 3). The random forest analyses were subsequently performed using Python software (version 3.7.1) to further confirm the utility of differential metabolites in distinguishing among HC and aMCI status.

## Results

### Demographic Differences Among HC, MCI and AD Groups

The demographic characteristics of study participants are detailed in Table 1. The age (HC: 62.61 ± 8.409, MCI: 68.22 ± 8.140, AD: 71.10 ± 8.899, *P* = 0.001), MMSE score (HC: 27.87 ± 1.792, MCI: 26.16 ± 2.567, AD: 18 ± 5.226, *P* < 0.001) and Montreal cognitive assessment (MoCA) score (HC: 26 ± 2.908, MCI: 20.33 ± 3.458, AD: 13.90 ± 5.413, *P* < 0.001) were significantly different among groups, while no significant differences in education level, gender ratio or apolipoprotein Eε4 (APOEε4) genotype were noted. The *post hoc* analysis of HC, MCI, and AD has been provided in the Supplementary Table 4.

**Table 1:** General characteristics and clinical data of participants.

	**HC** **(*n* = 23)**	**MCI** **(*n* = 45)**	**AD** **(*n* = 39)**	***p***
Age (years), mean (SD)	62.61 (8.409)	68.22 (8.140)	71.10 (8.899)	0.001^*^
Gender (male/female)	5/18	15/30	18/21	0.140
APOEε4 allele (absence/presence)	14/6	34/9	19/11	0.329
Education (years), mean (SD)	8.22 (5.143)	7.62 (3.557)	6.51 (3.872)	0.271
MMSE score, mean (SD)	27.87 (1.792)	26.16 (2.567)	18.00 (5.226)	<0.001^*^
MoCA score, mean (SD)	26.00 (2.908)	20.33 (3.458)	13.90 (5.413)	<0.001^*^

*One-way ANOVA analysis were used to examine the differences in the characteristics of HC, MCI and AD groups, and categorical data were compared using χ2 tests*.

* *Statistically significant differences (p < 0.05)*.

### Serum Untargeted Lipidomics Analysis of HC, MCI and AD Subjects

**Figure 2:**
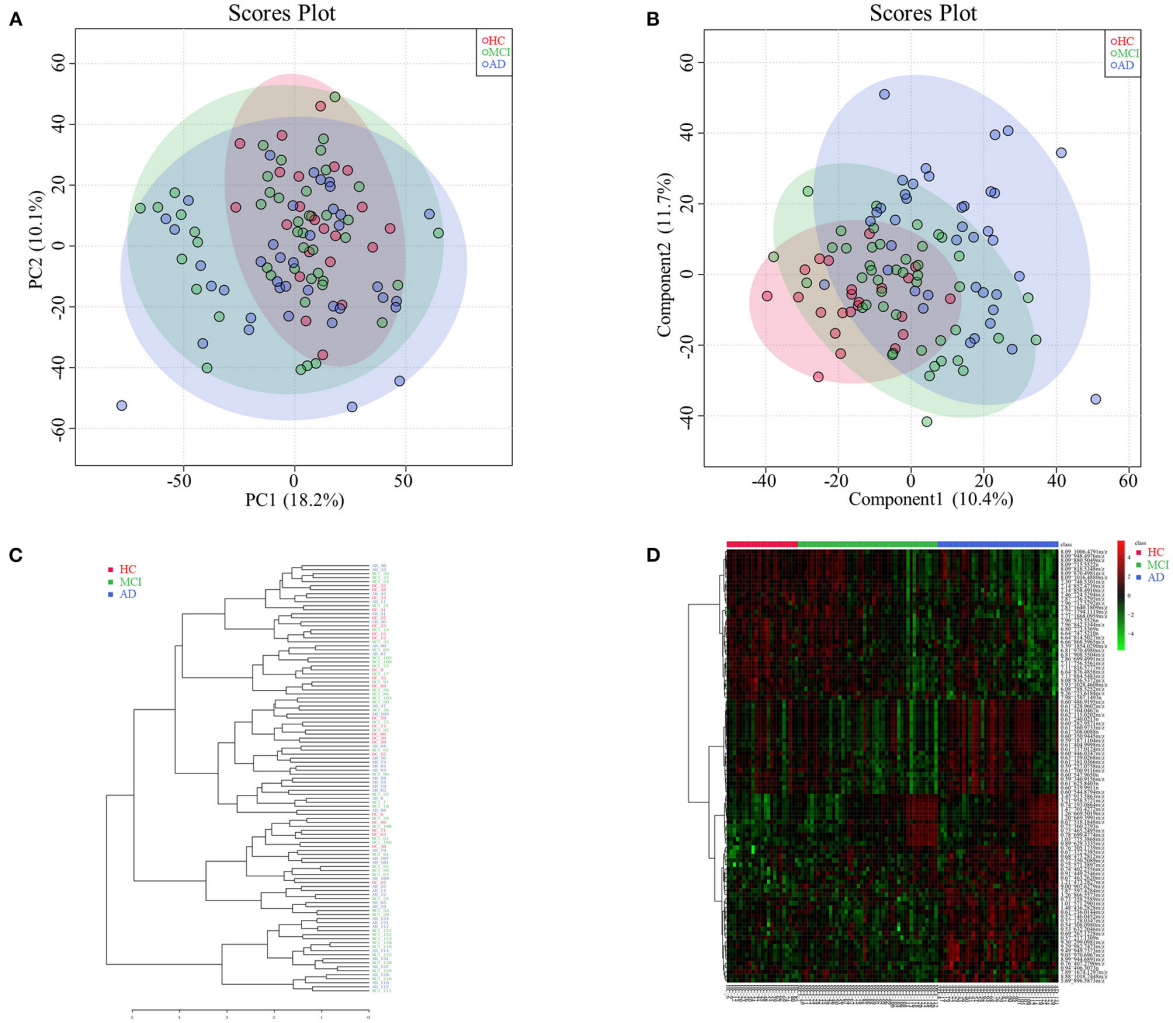
**(A)** Principal component analysis diagram of HC, MCI and AD comparison. **(B)** Partial least squares-discriminant analysis diagram of HC, MCI and AD comparison. **(C)** Dendrogram construction of HC, MCI and AD comparison. **(D)** Heatmap results of HC, MCI and AD comparison.

Among HC, MCI, and AD groups, 101 types of different retention time-mass charge ratio (RT-MZ) (*P* < 0.05) were obtained in NEG mode, among which were 4 types with *P* < 0.01. Thirteen RT-MZ types were significant in all 3 pairwise comparisons, while 9 types were significant in only one pairwise comparisons among the groups; namely between MCI and AD groups (Supplementary Table 5). Serum metabolome PCA and PLSDA revealed significant differences among all 3 groups; MCI group samples were midrange between the HC and AD groups (Figures 2A,B). Consistent with prior results, dendrogram construction revealed clustering of all 107 samples (Figure 2C).

Analysis of heatmap data of different metabolites (*P* < 0.05) revealed significant differences among HC, MCI and AD groups. Differential metabolites of MCI patients were found to be midrange between HC and AD groups (see Figure 2D for further subdivision). Analysis of serum untargeted metabolomic analysis of HC, MCI and AD patients in POS mode are shown in Supplementary Table 6.

### Serum Untargeted Lipidomics Analysis of aMCI and HC Subjects

**Figure 3:**
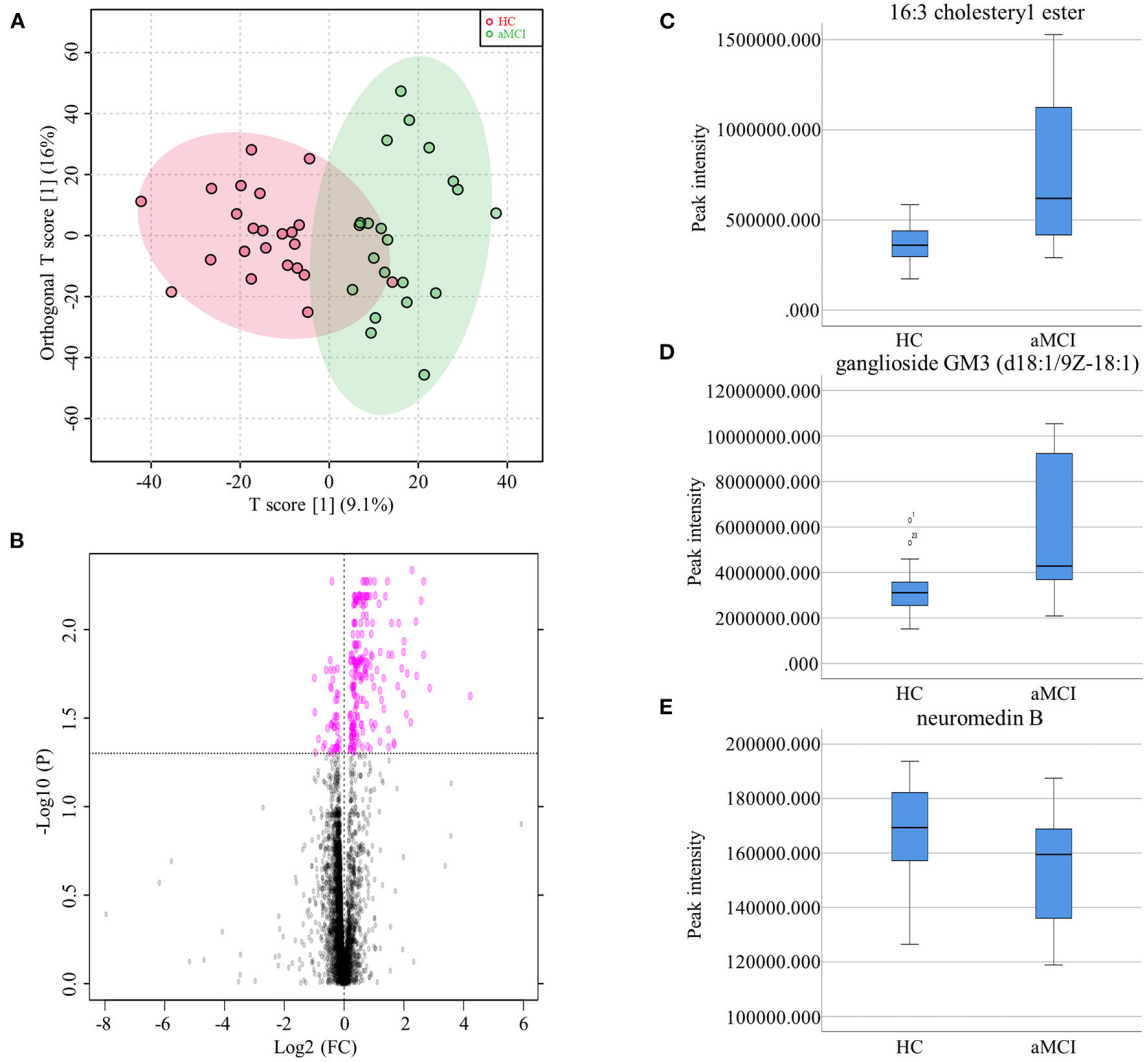
**(A)** Orthogonal partial least squares discriminant analysis diagram of HC and aMCI comparison. **(B)** Volcano Plots of HC and aMCI comparison. **(C)** Box plots showed the different peak intensity of 16:3 cholesteryl ester between HC and aMCI groups. **(D)** Box plots showed the different peak intensity of ganglioside GM3 (d18:1/9Z-18:1) between HC and aMCI groups. **(E)** Box plots showed the different peak intensity of neuromedin B between HC and aMCI groups.

The MCI group was further divided aMCI and naMCI subgroups and the serum metabolites were subsequently compared. No significant difference in either NEG or POS modes were noted. Next, serum metabolites of HC and aMCI patients were compared. The age (HC: 62.61 ± 8.409, aMCI: 69.57 ± 7.152, *P* = 0.005), MMSE score (HC: 27.87 ± 1.792, aMCI: 25.19 ± 2.839, *P* = 0.001) and MoCA score (HC: 26.00 ± 2.908, aMCI: 18.62 ± 3.025, *P* < 0.001) were significantly different among HC and aMCI groups, while patient sex, APOEε4 allele data, body mass index (BMI) and education level were not found to be significantly different (Table 2). To explore the potential biomarkers helpful in distinguishing HC from aMCI patients, we analyzed serum data with an orthogonal partial least squares discriminant analysis (OPLS-DA) in NEG mode. Findings revealed serum metabolites of these groups to significantly differ but at the same time partially overlap (Figure 3A). In total, 238 types of metabolites were obtained from comparison of HC and aMCI groups (*P* < 0.05) (Figure 3B). Here, we aimed to screen out RT-MZ data capable of distinguishing HC from aMCI as much as possible, but could not clarify all specific substances corresponding to RT-MZ. Of these 238 RT-MZ data, 24 corresponded with unique substances; after repetitions were removed, 20 substances were identified and divided into 6 categories: sterol lipids, sphingolipids, glycerophospholipids, fatty acids, saccharolipids and others (Table 3). The box plot reflects differences in 16:3 cholesteryl ester, ganglioside GM3 (d18:1/9Z-18:1) and neuromedin B in the HC and aMCI groups (Figures 3C–E). The peak intensity of 16:3 cholesteryl ester and ganglioside GM3 (d18:1/9Z-18:1) were higher in aMCI as compared to HC patients, while the peak intensity of neuromedin B was higher among HC subjects.

**Table 2:** General characteristics and clinical data of HC and aMCI patients.

	**HC** **(*n* = 23)**	**aMCI** **(*n* = 21)**	***P***
Age (years), mean (SD)	62.61 (8.409)	69.57 (7.152)	0.005^*^
Gender (male/female)	5/18	5/16	>0.99
APOEε4 allele (absence/presence)	14/6	13/6	>0.99
BMI(kg/m^2^), mean (SD)	23.17 (2.265)	23.55 (3.193)	0.651
Education (years), mean (SD)	8.22 (5.143)	8.24 (4.073)	0.988
MMSE score, mean (SD)	27.87 (1.792)	25.19 (2.839)	0.001^*^
MoCA score, mean (SD)	26.00 (2.908)	18.62 (3.025)	<0.001^*^

*Independent t-tests were used to examine the differences in the characteristics of the HC group and aMCI group, and categorical data were compared using χ2 tests*.

* *Statistically significant differences (p < 0.05)*.

**Table 3:** The significantly altered metabolites in the comparison of HC and aMCI.

**Metabolite**	***P*-value**
Sterol lipids	
16:3 Cholesteryl ester	0.0000275
1alpha-hydroxy-22-[3-(1-hydroxy-1-methylethyl)phenyl]-23,24,25.26,27- pentanorvitaminD3/1alpha-hydroxy-22-[3-(1-hydroxy-1-methylethyl)phen -yl]-23,24,25,26,27-pentanorcholecalciferol	0.00054943
Sphingolipids	
PE-Cer(d14:2(4E,6E)/16:0(2OH))	0.0000327
N-(tetradecanoyl)-deoxysphing-4-enine-1-sulfonate	0.0000358
Ganglioside GM3 (d18:1/9Z-18:1)	0.00030583
Glycerophospholipids	
LPIM1(19:0/0:0)	0.0000897
CL(8:0/11:0/18:2(9Z,11Z)/18:2(9Z,11Z))	0.0013938
PG(P-16:0/12:0)	0.0015922
PI(P-20:0/22:2(13Z,16Z))	0.0024846
Fatty Acyls	
IC202B	0.00012983
Lysine-containing siolipin	0.00033586
Saccharolipids	
DAT(19:0/25:0(2Me[S],3OH[S],4Me[S],6Me[S]))	0.0020767
Others	
Nonoxynol-9	0.0000601
2-Decaprenyl-6-methoxyphenol	0.00027562
Remikiren	0.00036897
Hydroxydestruxin B	0.00052018
Drotaverine	0.0006352
Neuromedin B	0.00074886

**Figure 4:**
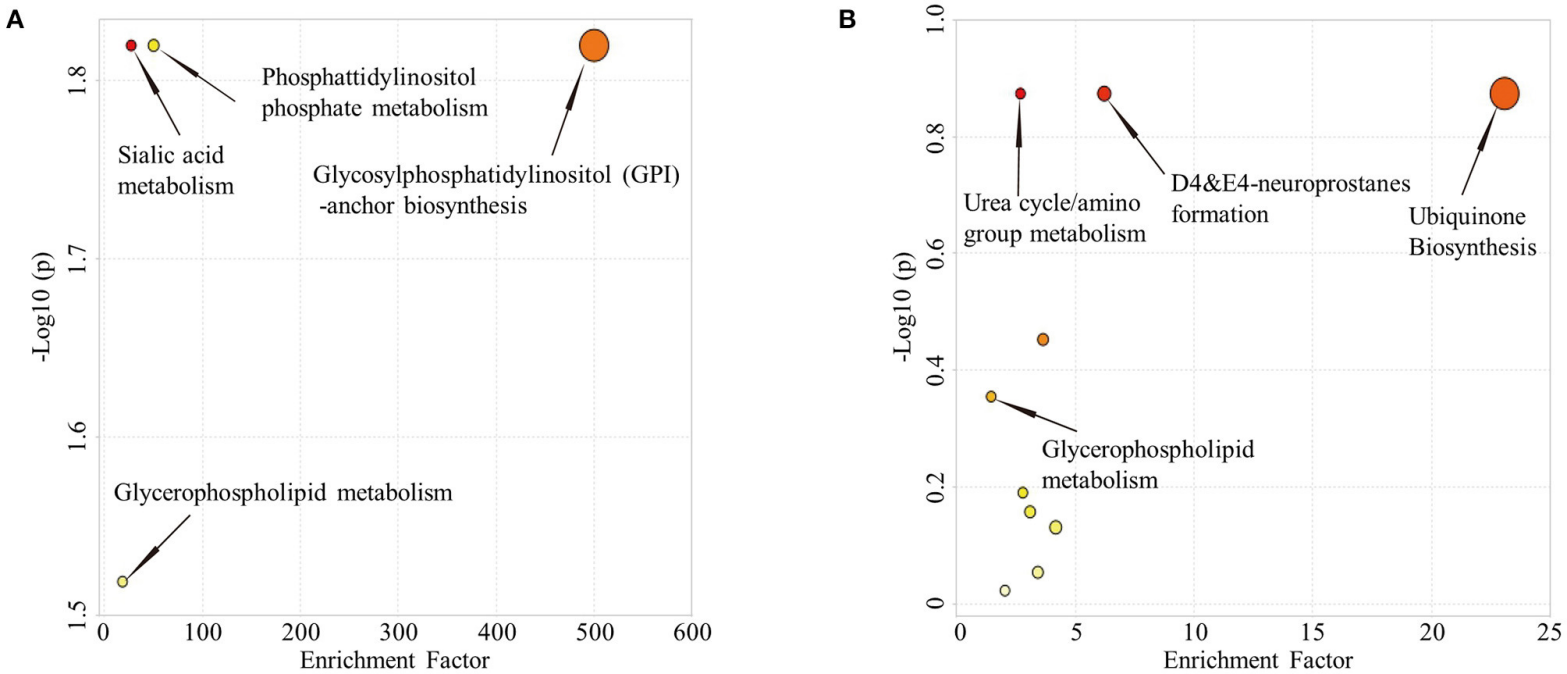
**(A)** Metabolic pathways from HC to aMCI. **(B)** Metabolic pathways from aMCI to AD.

Comparison of HC and aMCI groups revealed no significant findings in POS mode; Comparison of aMCI and AD groups revealed no significant findings in NEG mode. While in POS mode, comparison of aMCI and AD groups revealed 146 different RT-MZ (*P* < 0.05), of which 10 corresponded to unique substances (Supplementary Table 7). In both HC/aMCI and aMCI/AD group comparison, 16:3 cholesteryl ester was found. In order to explore the metabolic pathways that contribute to AD pathogenesis, we performed a metabolic pathway analysis using metabolites with *P* < 0.05 to compare HC and aMCI, as well as aMCI and AD patients. Comparison of HC and aMCI groups in NEG mode revealed 4 metabolic pathways differentiating these groups; namely sialic acid metabolism, phosphatidylinositol phosphate metabolism, glycosylphosphatidylinositol (GPI)-anchor biosynthesis, and glycerophospholipid metabolism. The latter three were the most significantly different (Figure 4A). Comparison of aMCI and AD groups in POS mode revealed 10 metabolic pathways differentiating HC and aMCI; among them ubiquinone biosynthesis, D4&E4-neuroprostane formation and urea cycle/amino group metabolism. These were found to exhibit the most significant differences (Figure 4B). Glycerophospholipid metabolism was a pathway common among HC/aMCI and aMCI/AD groups.

### Metabolites Differentiate HC and aMCI Subjects

**Figure 5:**
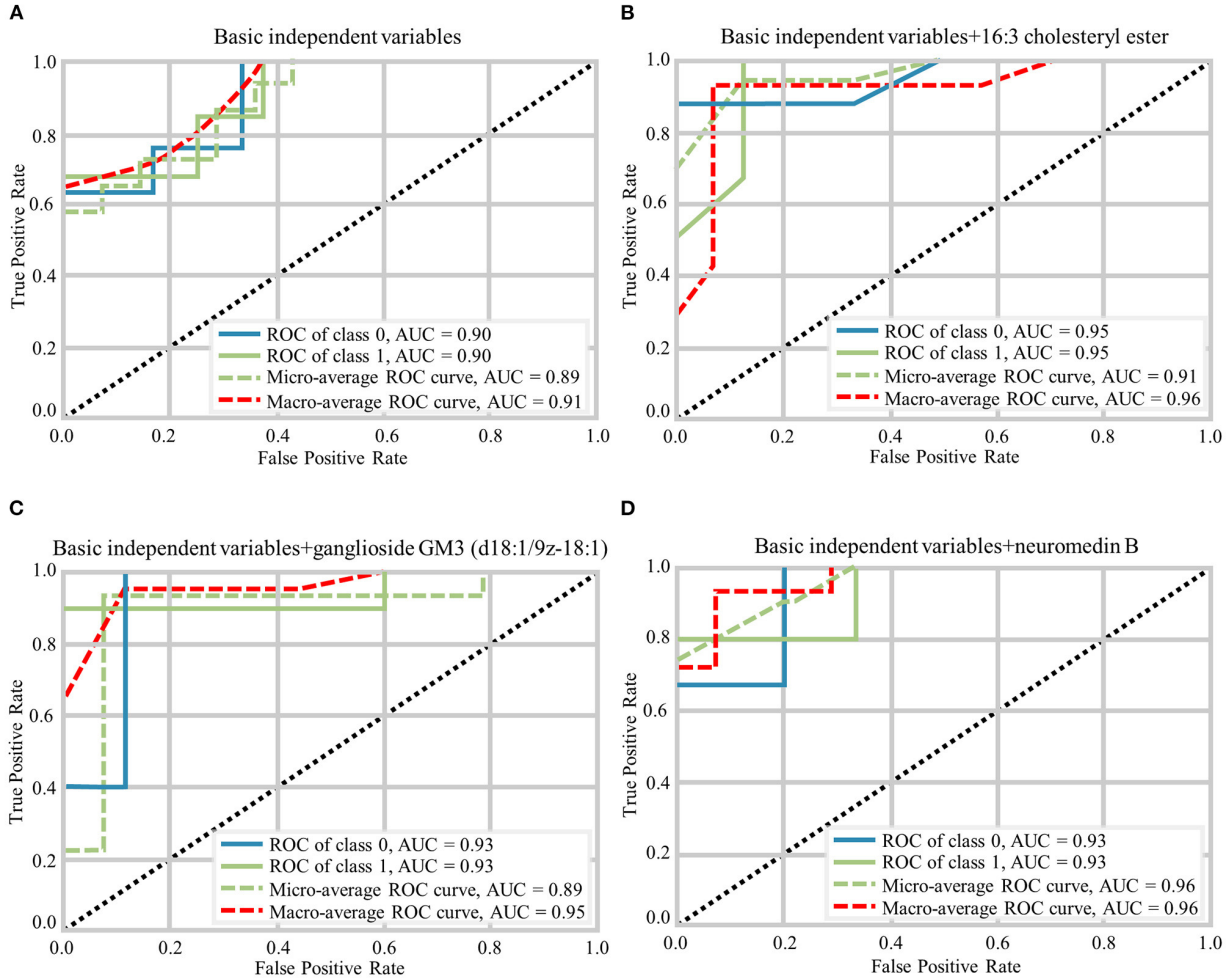
Different prediction models generated by Random Forest Classifier and the macro-average ROC curve considered as a reference. **(A)** The ROC curves of basic independent variables. **(B)** The ROC curves of basic independent variables+16:3 cholesteryl ester. **(C)** The ROC curves of basic independent variables+ganglioside GM3 (d18:1/9z-18:1). **(D)** The ROC curves of basic independent variables+neuromedin B.

**Figure 6:**
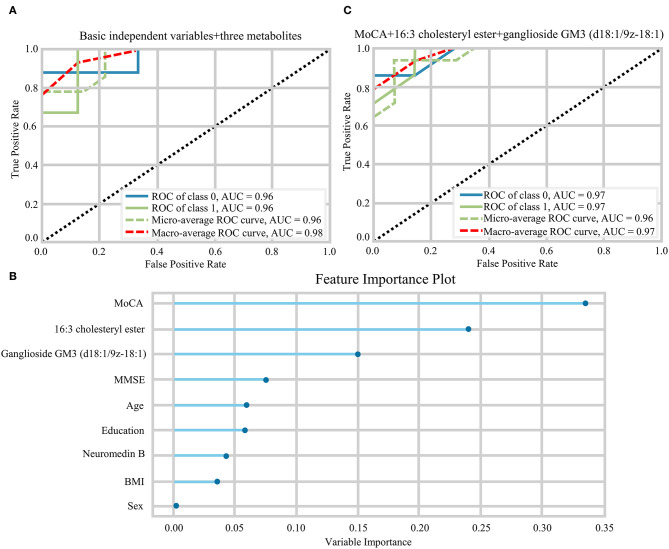
**(A)** The ROC curves of all independent variables. **(B)** Feature importance ranking of prediction model with all independent variables. **(C)** The ROC curves of MoCA+16:3 cholesteryl ester+16:3 cholesteryl ester.

The random forest analysis was used to further verify the usefulness of 16:3 cholesteryl ester, ganglioside GM3 (d18:1/9Z-18:1) and neuromedin B in distinguishing among HC and aMCI patients. Basic independent variables (age, sex, education level, BMI, MMSE, and MoCA data) were included in the Python random forest prediction model, and the prediction accuracy was found to be 0.91 (Figure 5A). The prediction accuracy was found to be 0.96, 0.95 and 0.96 when basic independent variables combined with 16:3 cholesteryl ester, ganglioside GM3 (d18:1/9Z-18:1) and neuromedin B, respectively (Figures 5B–D). When all independent variables including three metabolites were included in the random forest prediction model, prediction accuracy increased to 0.98 (Figure 6A). The predictive effect of aMCI increased by 0.07 after considering these 3 metabolites in combination. In addition, all independent variables among HC and aMCI groups were included in the model for feature importance ranking. Among these nine variables, MoCA score, 16:3 cholesteryl ester, and ganglioside GM3 (d18:1/9Z-18:1) had the greatest impact on the model, while gender had the least influence (Figure 6B). We combined MoCA score, 16:3 cholesteryl ester, and ganglioside GM3 (d18:1/9Z-18:1) to make a model and found that the predictive effect of aMCI was up to 0.97 (Figure 6C). These findings confirm that 16:3 cholesteryl ester, ganglioside GM3 (d18:1/9Z-18:1) and neuromedin B, especially the first two are important in distinguishing HC and aMCI subjects.

## Discussion

Here, we performed non-targeted lipidomics analysis to confirm differences in serum metabolites among HC, MCI, and AD groups, and compared serum differential metabolites of HC and aMCI subjects in order to obtain lipid biomarkers capable of discriminating aMCI cases from HC subjects and applicable even in the early prediction of AD. Firstly, we found significant differences in serum metabolites among HC, MCI and AD groups; 96 different RT-MZ were obtained in NEG mode. Furthermore, 238 different RT-MZ and 20 metabolites were obtained in NEG mode through the comparison of HC and aMCI. Secondly, metabolic pathway analysis suggested glycerophospholipid metabolism to be a common pathway among HC/aMCI and aMCI/AD subjects, underscoring how glycerophospholipid metabolism plays an important role in the progression of AD, and that 16:3 cholesterol ester is closely related to glycerophospholipid metabolism. Thirdly, random forest analysis revealed that age, sex, education level, BMI, MMSE, and MoCA–taken togethe-are capable of predicting aMCI at an accuracy of 0.91, while combination with 16:3 cholesteryl ester, ganglioside GM3 (d18:1/9z-18:1) and neuromedin B increased predictive accuracy to 0.98. Three variables that had the greatest impact on the model were selected to set a new predication model, whose prediction performance was roughly equivalent to that of the model with all independent variables.

Here, 16:3 cholesteryl ester was the only metabolite found in both HC/aMCI and aMCI/AD comparisons. Its peak intensity increased with the severity of AD, underscoring its utility in AD staging and monitoring. Ganglioside GM3 (d18:1/9z-18:1) and neuromedin B were found in the HC/aMCI comparison, indicating their capacity to discriminate aMCI cases from HC subjects. These three biomarkers improve the predictive accuracy for aMCI individuals and the model with MOCA score, 16:3 cholesteryl ester, and ganglioside GM3 (d18:1/9Z-18:1) can be independently used for early AD prediction.

Previous studies have revealed high cholesterol levels to associated with an increased risk of developing AD both in animal and human studies (19, 20). Normally, due to the blood brain barrier, cerebral cholesterol is produced almost entirely via de novo synthesis (21). Neuronal cellular machinery responds to an excess of cholesterol in a variety of ways, such as esterification and subsequent intracellular storage in lipid droplets, direct excretion through the ATP binding cassette transporters (22, 23) or conversion to 24(S)-hydroxycholesterol (24). The 24(S)-hydroxycholesterol can exit the brain either by diffusion or by organic anion transport across the barrier (25).

Possible mechanisms of cognitive dysfunction caused by excessive cholesterol in the brain are likely related to oxidative stress and Aβ production. Oxidative stress disrupts acyl-CoA:cholesterol acyltransferase (26); inhibition of acyl-CoA:cholesterol acyltransferase activity causes a significant reduction in cholesteryl esters, amyloid level, and brain amyloid plaques (27–29). Aβ production, metabolism and aggregation also depend on lipid rafts (30). In addition, elevated cholesterol levels may increase 24 hydroxycholesterol in the brain, which was suggested to be neurotoxic and to potentiate Aβ toxicity (31).

Although it remains unclear whether blood lipid changes or AD occurs first, changes in blood lipids almost certainly reflect illness. In recent years, studies have found long chain cholesterol esters to be associated with AD, in particular the cholesterol esters 32:0, 34:0, 34:6, 32:4, 33:6, and 40:4. The plasma concentrations of these molecules are highest in HC, lower in MCI patients and lowest in the setting of overt AD (32). Our findings, however, revealed that peak levels of 16:3 cholesterol esters in serum were lowest in HC, higher in MCI patients and highest in AD patients. Elevated levels of these molecules in aMCI patients likely associates with cholesterol esters synthesized from 24 hydroxycholesterol excreted from the central nervous system.

Ceramides (Cer) are the simplest sphingolipids. Normally, ganglioside metabolism includes both a-series (Cer→ GlcCer→ LacCer→ GM3→ GM2→ GM1→ GD1a) and b-series (Cer→ GlcCer→ LacCer→ GM3→ GD3→ GD2→ GD1b→ GT1b) metabolism. Complex gangliosides are the predominant form of gangliosides expressed within the healthy adult brain; simple gangliosides are only expressed in small quantities (33). Previous studies have found simple gangliosides, in particular GM3, to be consistently increased in the brain of AD patients, while complex gangliosides (GT1b, GD1b, GD1a and GM1) appeared to be uniformly decreased (34–36). The cause of this increased proportion of GM3 in the AD brain is likely associated with astrogliosis (34–36) and the enhancement of the catabolic pathway of more complex gangliosides (37). Indeed, GM3 has been suggested to upregulate pro-apoptotic signaling pathways as well-inhibit vascular endothelial growth factor receptor activity, thus leading to toxicity when accumulated in neurons (38, 39). Moreover, Aβ was reported to have a high affinity for interaction with gangliosides (40). This results in a change of ganglioside-Aβ structural conformations, leading to aggregation of Aβ (41–43). However, the reduced neuroprotective effect of GM1 also likely plays a role (44, 45).

Unfortunately, available literature mainly reports elevated GM3 levels in the AD brain; studies exploring the elevation of blood GM3 levels are scarce. Here, serum GM3 levels were found to be increased in aMCI patients as compared to HC, consistent with prior literature that reported cerebral GM3 levels to be increased in AD patients (34–36). These findings further suggest that changes in serum gangliosides levels indeed reflect early cerebral lesion formation in AD.

Gastrin-releasing peptide and neuromedin B are both members of the bombesin-like peptide family. Previous studies reported that locally produced neuromedin peptides and/or peptide fragments can be transported throughout the entire body, including transport across the blood brain barrier (46). Early studies reported that either systemic administration of gastrin-releasing peptide receptor agonists or infusion of the agonist-Bombesin into the hippocampus improves memory retention in rodent models (47, 48). Possible mechanisms of this phenomenon include gastrin-releasing peptide modulated neurogenesis and neuronal development, thus contributing to hippocampal circuit function (49). Moreover, gastrin-releasing peptide and neuromedin B were reported to restores impaired synaptic plasticity and were able to elevate expression of synaptic proteins, synaptophysin and Ca2+/calmodulin dependent protein kinase II, all of which play pivotal roles in synaptic plasticity (50). To date, few studies exploring the association between neuromedin B and dementia exist in literature. Only Yang et al., verified that gastrin-releasing peptide and neuromedin B substantially improve spatial learning and memory abilities in a rat model of vascular dementia (50). Since these molecules are both homologous substances, gastrin-releasing peptide can be postulated to exert effects similar to neuromedin B. Previous studies have not found a relationship between serum neuromedin B levels and MCI or AD status; we here first report that serum neuromedin B levels effectively distinguish HC from aMCI patients.

In summary, our research reveals that 16:3 cholesteryl ester, ganglioside GM3 (d18:1/9z-18:1) can be effectively used as biomarkers in the early clinical prediction of aMCI or AD. We found severity of dementia to positively correlate to serum 16:3 cholesteryl ester and ganglioside GM3 levels. We also found that lower levels of neuromedin B were expressed in the serum of aMCI patients as compared to HC subjects. Although glycerophospholipid metabolism plays an important role in the progression of AD, the specific mechanisms by which the aforementioned molecules influence dementia remain unclear. In addition, due to the deficiency of small sample size and cross-sectional study, the conclusions obtained are relatively preliminary, which requires further repeated studies evaluating a larger number of subjects and extending the follow-up time to investigate the potential clinical utility of metabolic biomarkers in the diagnosis and treatment of both aMCI and AD.

## Limitations

This study had several limitations. First, only 107 participants (including 21 aMCI participants) in total were recruited. This relatively small sample may reduce the credibility of our conclusions. Second, study participants stopped taking relevant drugs for 1 month prior to blood sample collection. Ideally, study participants should stop taking medications at least 3 months prior to study commencement to ensure that no residual drug levels remain in the body. We thus could not exclude any potential effects of certain medications on serum lipidomics profiling, nor the influence of different dietary habits among study participants. Third, if the obtained biomarkers could be compared to others currently known, such as p-tau-181, their efficacy would be better reflected. In addition, the effectiveness of biomarkers detailed in our study in distinguishing HC and aMCI should be further compared with cognitive assessment scores. Finally, our comparison of aMCI and naMCI status revealed no significant differences. It would be meaningful for early and accurate prediction of AD if future studies evaluate different metabolites among aMCI and naMCI sub-groups. While our results are novel and promising, these aforementioned limitations should be addressed in future studies to fully validate clinical application of the biomarkers we detailed.

## Data Availability Statement

The original contributions presented in the study are included in the article/Supplementary Material, further inquiries can be directed to the corresponding authors.

## Ethics Statement

The studies involving human participants were reviewed and approved by First Affiliated Hospital, School of Medicine, Zhejiang University. The patients/participants provided their written informed consent to participate in this study.

## Author Contributions

GP and LZ conceived and designed the project. LZ wrote the manuscript with inputs from YS. JY, FH, and MH provided guidance on data analysis. LL, FH, YLin, XL, YLiu, and BL helped to revise the manuscript. All authors contributed to the article and approved the submitted version.

## Conflict of Interest

The authors declare that the research was conducted in the absence of any commercial or financial relationships that could be construed as a potential conflict of interest.

## Publisher's Note

All claims expressed in this article are solely those of the authors and do not necessarily represent those of their affiliated organizations, or those of the publisher, the editors and the reviewers. Any product that may be evaluated in this article, or claim that may be made by its manufacturer, is not guaranteed or endorsed by the publisher.

## Supplementary Material

Supplementary Table 1. The metabolite identification of RT-MZ in NEG mode.

Supplementary Table 2. The metabolite identification of RT-MZ in POS mode.

Supplementary Table 3. Prediction results of multiple machine learning models.

Supplementary Table 4. The post hoc analysis of HC, MCI, AD groups.

Supplementary Table 5. The RT-MZ obtained from the comparison of HC, MCI and AD in NEG mode.

Supplementary Table 6. The RT-MZ obtained from the comparison of HC, MCI and AD in POS mode.

Supplementary Table 7. The RT-MZ obtained from the comparison of aMCI and AD in POS mode.

## Abbreviations

AD: Alzheimer's disease
MCI: mild cognitive impairment
aMCI: amnestic mild cognitive impairment
naMCI: non-amnestic mild cognitive impairment
HC: healthy controls
BMI: body mass index
MMSE: mini-mental State Examination
MOCA: Montreal Cognitive Assessment
AUC: area under the curve
PCA: principal component analysis
OPLS-DA: orthogonal partial least squares-discriminant analysis
APOEε4: apolipoprotein Eε4
RT-MZ: retention. time-mass charge ratio
Cer: Ceramides.
