# Supplementary material for: Serum Metabolites Differentiate Amnestic Mild Cognitive Impairment From Healthy Controls and Predict Early Alzheimer's Disease via Untargeted Lipidomics Analysis
Source: Front Neurol. 2021 Aug 2;12:704582. doi: 10.3389/fneur.2021.704582 (PMC8365883; doi:10.3389/fneur.2021.704582)
Supplement: Supplementary Table 3. — Prediction results of multiple machine learning models. [file Table_3.docx]

| **Model** | **Accuracy** | **AUC** | **Recall** | **Prec.** | **F1** | **Kappa** | **MCC** | **TT (Sec)** |
| --- | --- | --- | --- | --- | --- | --- | --- | --- |
| **dt** | Decision Tree Classifier | 0.9667 | 0.9750 | 1.0000 | 0.9500 | 0.9667 | 0.9400 | 0.9500 |
| **rf** | Random Forest Classifier | 0.9667 | 0.9500 | 1.0000 | 0.9500 | 0.9667 | 0.9400 | 0.9500 |
| **ada** | Ada Boost Classifier | 0.9667 | 0.9750 | 1.0000 | 0.9500 | 0.9667 | 0.9400 | 0.9500 |
| **gbc** | Gradient Boosting Classifier | 0.9667 | 0.9750 | 1.0000 | 0.9500 | 0.9667 | 0.9400 | 0.9500 |
| **xgboost** | Extreme Gradient Boosting | 0.9667 | 0.9750 | 1.0000 | 0.9500 | 0.9667 | 0.9400 | 0.9500 |
| **et** | Extra Trees Classifier | 0.9333 | 1.0000 | 0.9000 | 0.8500 | 0.8667 | 0.8400 | 0.8500 |
| **catboost** | CatBoost Classifier | 0.9333 | 0.9500 | 1.0000 | 0.9167 | 0.9467 | 0.8400 | 0.8500 |
| **lr** | Logistic Regression | 0.9000 | 0.9500 | 0.8500 | 0.8500 | 0.8333 | 0.7800 | 0.8000 |
| **nb** | Naive Bayes | 0.9000 | 1.0000 | 0.8500 | 0.8500 | 0.8333 | 0.7800 | 0.8000 |
| **ridge** | Ridge Classifier | 0.9000 | 0.0000 | 0.8500 | 0.8500 | 0.8333 | 0.7800 | 0.8000 |
| **svm** | SVM - Linear Kernel | 0.8667 | 0.0000 | 0.9500 | 0.8500 | 0.8833 | 0.7300 | 0.7500 |
| **lda** | Linear Discriminant Analysis | 0.8667 | 0.9500 | 0.9000 | 0.9167 | 0.8800 | 0.7200 | 0.7500 |
| **knn** | K Neighbors Classifier | 0.7667 | 0.9000 | 0.6500 | 0.7500 | 0.6667 | 0.5100 | 0.5500 |
| **qda** | Quadratic Discriminant Analysis | 0.7000 | 0.7500 | 0.9500 | 0.7167 | 0.7633 | 0.4800 | 0.5000 |
| **lightgbm** | Light Gradient Boosting Machine | 0.3333 | 0.5000 | 0.5000 | 0.1667 | 0.2500 | 0.0000 | 0.0000 |

**Supplementary Table 3:** Prediction results of multiple machine learning models

**Abbreviations:**

AUC: Area Under Curve; Prec: Precision; F1: F1-Score, an index considering both the accuracy rate and recall rate; Kappa: an index used to evaluate consistency; MCC; Matthews correlation coefficient, tthe value of 1 indicates a perfect prediction, and value of 0 indicates that it is not better than random prediction.
